# Reprogramming Inflammatory Macrophages with Specialized Pro-Resolving Lipid Mediators: A Novel Immunotherapeutic Strategy for Asthma

**DOI:** 10.3390/biomedicines14071432

**Published:** 2026-06-24

**Authors:** Ruchita Tanu, Ashraf A. Qurtam, Gagan Prakash, Anis Ahmad Chaudhary, Nadeem Raza, Pushpender K. Sharma, Sudarshan Singh Lakhawat, Tejpal Yadav, Monika Kaushik, Vikram Kumar

**Affiliations:** 1 Amity Institute of Biotechnology, Amity University Rajasthan, Jaipur 303002, Rajasthan, India; 2 Department of Biology, College of Science, Imam Mohammad Ibn Saud Islamic University (IMSIU), Riyadh 11623, Saudi Arabia; 3 Department of Chemistry, College of Science, Imam Mohammad Ibn Saud Islamic University (IMSIU), Riyadh 11623, Saudi Arabia; 4 Amity Institute of Pharmacy, Amity University Rajasthan, Jaipur 303002, Rajasthan, India; 5 School of medical and allied sciences, K R Mangalam University, Sohna Road, Gurugram 122103, Haryana, India

**Keywords:** specialized pro-resolving lipid mediators (SPMs), macrophage reprogramming, inflammation resolution, asthma immunomodulation, personalized resolution medicine

## Abstract

Asthma is defined as a chronic airway inflammatory disorder with over-activation of the immune system accompanied by the inability to resolve inflammation. SPMs are novel potent lipid mediators that play an important role in maintaining inflammation homeostasis and macrophages’ functional plasticity. This review will look into the potential function of SPM-programmed macrophage reprogramming as a novel therapeutic strategy for asthma. Unlike current anti-inflammatory treatments, which only focus on suppressing inflammation, SPMs can actively drive the inflammation resolution phase by promoting efferocytosis and wound healing while maintaining the defense against infection. In experimental asthma animal models, lipoxins, resolvins, protectins, and maresins have been demonstrated to alleviate inflammation and airway hyperresponsiveness, shift macrophages towards pro-resolving phenotypes and thus facilitate the resolution process. Levels of some SPM subclasses were found to be reduced in severe or uncontrolled asthmatics, indicating defective resolution pathways may contribute to asthma persistence. The mechanisms include down-regulation of pro-inflammatory cytokines, alteration of macrophage phenotype, improvement of immune homeostasis in the airway milieu, etc. These molecules have become highly promising therapeutic agents after the development of metabolically stable analogs, receptor-targeted agonists, and an improved delivery system. Multi-omics studies coupled with patient stratification based on biomarkers will potentially help in the future to develop personalized resolution-based therapy, in particular for those steroid-resistant and non-type 2 asthmatics. Nevertheless, the evidence provided so far is mainly preclinical; more challenges in terms of pharmacokinetics, formulation and formulation development, regulatory agency approval, and clinical validation remain and will be overcome through further studies, thus warranting investigation into SPM-mediated strategies for asthma and other chronic inflammatory diseases.

## 1. Introduction

More than 300 million people around the world have asthma, and asthma is a serious, manageable illness. In North America alone, more than 30 million people have asthma and are experiencing respiratory problems. Those with asthma face significant health costs and challenges, as does society as a whole [1]. Asthma may arise in anyone at any age, and periodic changes are possible with asthma. Asthma will exhibit unpredictably at multiple times and in various ways (displayed by individual patients differently). For many decades, provocation by a Th2-mediated process was understood to be the sole mediator of asthma, but updated research indicates there are numerous etiology/endotypes for asthma; Asthma, therefore, will include complex immune systems, epithelial cell differences, and significantly different metabolic processes as well as distinct airway structural differences in patients [2,3,4].

Currently, most treatments utilize medications such as inhaled corticosteroids (ICS), β_2_-agonists (β_2_-A), and leukotriene inhibitors (LT Mod) to manage inflammation caused by asthma and to help patients manage their symptoms. Although these therapies adequately address the needs of many asthma patients, there continues to be a large number of patients with uncontrolled asthma that cannot be adequately treated or controlled with ICS, β_2_-A, or LT Mod. This is more common among patients who suffer from severe asthma or those who have non-2 asthma [5,6]. In addition, the previously mentioned therapies mainly block inflammatory pathways; therefore, the immune system’s response will still be imbalanced and will not be able to repair/improve the lining of the airways/tissues. Those patients who have preexisting inflammatory conditions in the airways, multiple increases and/or continual development of the airway will develop more frequently from other kinetic processes that have occurred despite the repeated immunosuppressive treatment [7,8].

Despite rapid advances in IgE-, IL-5-, IL-5R-, IL-4R- and TSLP-directed biologics that have successfully translated to clinical improvement in certain patients with type 2 asthma, large groups of patients remain poorly controlled despite therapy. There is variability in clinical response, and patients with non-type 2 asthma, neutrophilic inflammation, and corticosteroid resistance appear to respond poorly to available biologics. Lastly, current biologic therapies only antagonize key inflammatory pathways but do not restore innate mechanisms responsible for the resolution of inflammation and tissue homeostasis; new therapies are required to combat both active inflammation and failure of resolution [9].

The increasing awareness of the failure of resolution of inflammation as a critical component of chronic airway inflammation has led to interest in specialized pro-resolving mediators (SPMs), the endogenous family of lipid mediators that participate in active termination of inflammation and return of tissue homeostasis [10].

In a normal person’s body, when they are exposed to a potentially harmful agent, their acute inflammatory response will last for a short time and will be resolved in an orderly manner as it will follow a specific pattern instead of just stopping. The process of resolution is accomplished through the action of specialized pro-resolving mediators (SPMs)—a class of lipid-derived substances composed of lipoxins, resolvins, protectins and maresins [11]. SPMs are created when polyunsaturated fats (PUFAs) break down into big fatty acids into small ones. They do so through special types of G-protein coupled receptors (such as ALX/FPR2, ChemR23, GPR18, and GPR37). The main difference between an anti-inflammatory drug and an SPM is that anti-inflammatories shut down the immune system response, but SPMs will enhance how the body responds with respect to healing (e.g., efferocytosing) and returning the immune system to its normal state (e.g., restoring homeostasis) [12,13].

Recent research indicates that asthma is not only due to a hyperactive immune system, but also that humans cannot properly resolve inflammation after allergic reactions, which are related to allergies. The decreased ability to resolve inflammation results from low levels of endogenous specialized pro-resolving mediators (SPM); issues with the functioning of the cell receptors; ineffective macrophages that cannot effectively clear away dead cells; an increase in NF-κB and MAPK signaling pathways; and other factors that lead to ongoing airways inflammation and injury. Macrophages are important regulators of the resolution versus the continuation of inflammation [14]. At the forefront of the inflammatory and resolution processes are macrophages. The shift in macrophage phenotypes from pro-inflammatory toward pro-resolving and tissue-repairing stages is critically important for adequate efferocytosis and removal of inflammatory waste products and the return of the airway to homeostasis. Accumulating data are increasingly implicating diminished macrophage plasticity in the chronicity of inflammation and steroid resistance, as well as airway remodeling in severe asthma. Any failure for the macrophages to change from a pro-inflammatory to a pro-resolution state is a significant pathological finding associated with the natural progression of asthma [15,16].

This understanding has completely shifted our approach to treating inflammation from a purely therapeutic basis (i.e., therapeutic resolution) to one that includes enhancing the ability of the body to resolve inflammation as well. Ideally, this means restoring normal endogenous pro-resolution pathways instead of simply blocking pro-inflammatory mediators. With regard to this latter goal, we believe that resolution pharmacology for steroid-refractory asthma may lead to a decrease in airway remodeling, less tissue destruction, and ultimately modify asthma long-term [17,18].

However, while it has become increasingly clear that impaired inflammatory resolution contributes to asthma pathophysiology, the precise molecular mechanisms underlying SPM regulation of macrophage plasticity and re-establishment of airway homeostasis are still largely undefined. Moreover, although many preclinical findings have suggested the pro-resolving potential of SPMs in experimental asthma models, to date, these results have failed to translate to successful clinical applications. It is essential that these knowledge gaps are filled in order to establish resolution-based therapeutic strategies for the treatment of difficult asthma conditions such as severe, steroid-resistant and non-type 2 disease, overcoming many of the limitations of conventional anti-inflammatory approaches [15,19,20].

In this review, we will evaluate the significance of specific SPMS in controlling macrophage plasticity and resolving inflammation in asthma. Special attention is paid to the mechanisms through which SPMS reprogram inflamed macrophages and restore immune balance in the airways and whether these pro-resolving signals might be the answer to the failings of existing therapies. The translational potential, the difficulties in delivering and developing pharmacologically, and the therapeutic opportunities that lie ahead for the resolution-based immunotherapies in asthma.

## 2. Immunopathogenesis of Asthma

### 2.1. Cellular and Molecular Mechanisms

**Figure 1:**
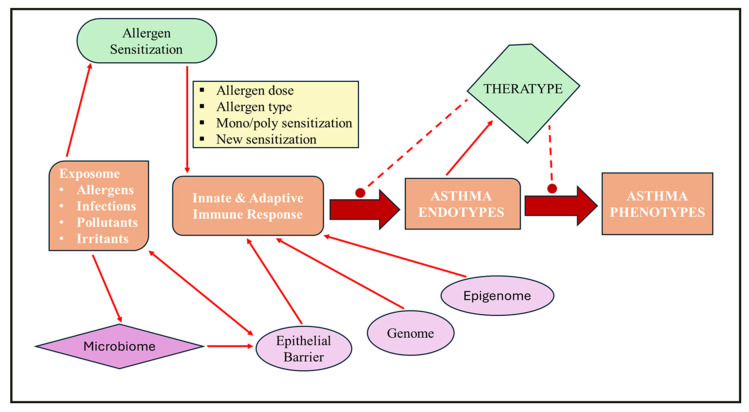
Asthma Immunopathogenesis and Theratypes. A schematic illustration of the multi-factorial and complex manner in which asthma develops and varies among individuals. Environmental exposures (exposome), such as allergens, infections, air pollution, and other irritants, all result in the initiation of allergic sensitization by the body, which stimulates both innate and adaptive immune responses of the body. The type and degree of allergic sensitization will be influenced by allergen dose, allergen type, whether the individual is mono- or polysensitized, and whether they have experienced new allergen sensitization events. Host factors, such as the microbiome, epithelial barrier integrity, genome, and epigenome, all play a role in modulating the immune system and contributing to the disease process. As a result of these environmental and host interactions, specific types of asthma endotypes emerge, which can then be expressed as asthma phenotypes that can be clinically observed. Many treatments (therapies) are becoming more dependent on each individual’s respective asthma endotype to provide precise treatment for the individual with asthma.

Asthma is classified as a chronic inflammatory disease of the airways that can cause varying degrees of airway obstruction, as well as increased sensitivity of the airways to non-infectious stimuli and continued dysregulation of the immune response. Activation of asthma is the result of an interplay between airway structure (cells) and cells of the immune system through stimulation by environmental threats (i.e., allergens, air pollution, and respiratory pathogens) as depicted in Figure 1. Typically, the airway epithelium serves as the initial immune sensing site for the detection of environmental threats through its production of alarmins (e.g., IL-25, IL-33, and TSLP), which condition downstream responses like inflammation [21,22].

**Figure 2:**
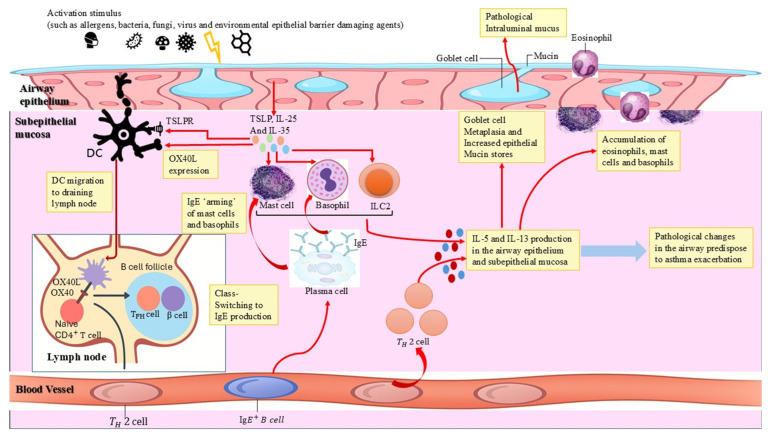
Asthma Immune Mechanisms: This schematic diagram illustrates the cellular and molecular mechanisms driving asthma pathogenesis. Environmental activation stimuli such as allergens, bacteria, fungi, viruses, and epithelial barrier-damaging agents trigger airway epithelial cells and dendritic cells (DCs). Activated DCs migrate to lymph nodes, where interaction with naive CD4+ T cells via OX40-OX40L signaling induces differentiation into follicular helper T cells (T_FH) and Th2 cells. Th2 cells and innate lymphoid cells type 2 (ILC2) produce cytokines IL-4, IL-5, and IL-13, leading to B cell class switching and IgE production by plasma cells. IgE “arms” mast cells and basophils, sensitizing them for allergen-induced activation. IL-5 mediates eosinophil recruitment and activation, causing their accumulation alongside mast cells and basophils in the subepithelial mucosa. IL-13 promotes goblet cell metaplasia and increased mucin production, resulting in pathological intraluminal mucus. These immune and epithelial changes synergize to induce airway inflammation, remodeling, and hyperresponsiveness characteristic of asthma, predisposing to exacerbations and airway obstruction.

The mediators of epithelial tissue activate dendritic cells and polarize CD4(+) T cells from a naïve state to a Th2-like state, which has been shown to increase the production of IL-4, IL-5, and IL-13, leading to the development of eosinophilic inflammation, mucus production, and airway hyperresponsiveness [23,24]. Macrophages not only regulate inflammation by acting as antigen-presenting cells and producing cytokines, but they also play an important role in the regulation of airway inflammation by removing apoptotic bodies from the airways via a process called efferocytosis. Dysregulation of macrophage polarization and impairment of efferocytosis are associated with the persistence of inflammation and impaired resolution of inflammation in patients with chronic asthma. The persistence of prolonged inflammatory signals ultimately leads to airway remodeling, which includes goblet cell hyperplasia, subepithelial fibrosis, and smooth muscle hypertrophy [25]. Figure 2 illustrates the key cellular and molecular pathways involved in asthma immunopathogenesis [26].

### 2.2. Environmental Activation and Allergen Sensitization

Environmental triggers such as allergens, respiratory viruses, pollutants, and chemical irritants prompt the initiation of asthma by disturbing the integrity of the airway epithelium and activating innate immune signaling pathways [27,28] as depicted in Figure 1. The release of the alarmins (e.g., thymic stromal lymphopoietin, IL-25, IL-33) by the airway epithelium in response to an injury initiates the activation of dendritic cells, innate lymphoid cells type 2, and macrophages [21,28,29]. The interactions of these early interacting cells lead to a Th2-dominated inflammatory response characterized by the presence of eosinophils, the production of cytokines, and hyperresponsiveness of the airway [28,29,30]. In addition, ongoing exposure to environmental agents exacerbates oxidative stress and inhibits resolution pathways, which contribute to chronic airway inflammation and immune dysregulation in asthma [27,30].

### 2.3. The Th2 Cytokine Milieu and Effector Cell Recruitment

The development of asthma is greatly affected by Th2 cells and group 2 innate lymphoid cells (ILC2s) due to their ability to produce IL-4, IL-5, and IL-13. Together, these interleukins stimulate the production of IgE, the eosinophil-associated inflammation of the lung, excessive mucus production, and an excessive airway response to allergen exposure [31,32,33]. After eosinophils are recruited to the lung in response to inflammatory cytokines, they degranulate and expel cytotoxic granule proteins, reactive oxygen species, and lipid mediators that amplify epithelial damage and increase inflammatory signaling. Mast cells and basophils contribute to airway obstruction by releasing mediators such as histamine and leukotrienes [34,35,36,37]. Eosinophils, mast cells, and basophils further stimulate the recruitment of additional eosinophils, which causes a persistent inflammatory response and leads to airway remodeling, including goblet cell hyperplasia, subepithelial fibrosis, and hypertrophy of the smooth muscle in the airway. Additionally, aberrant macrophage responses and poor clearance of apoptotic inflammatory cells also promote chronic airway inflammation and faulty resolution processes in asthma [15,38].

### 2.4. Epithelial and Mucosal Remodeling

In asthma, the damaging effects of ongoing inflammation in the airways create long-term, permanent changes in airway structure and function. Three hallmark features of airway remodeling associated with persistent airway inflammation in asthma are: (1) deterioration of airway epithelial function, (2) development of subepithelial fibrosis, and (3) excessive production of mucus by the airways, all leading to airway obstruction and worsening lung function [31,39,40]. Macrophages play a key role in modulating the processes of structural lung remodeling in asthma by releasing cellular signals (cytokines/growth factors such as TGF-beta) and proteins (matrix-modifying enzymes) that promote fibroblast growth and extracellular matrix (ECM) deposition [41]. Abnormalities in macrophage activation and impairment of normal macrophage inflammatory response mechanisms can also contribute to persistent damage to airway tissue and impaired repair of airway epithelium, which may factor into the development of more severe and persistent forms of asthma [36,42].

### 2.5. The Role of Macrophages in Asthma

The roles of macrophages as major immune cell types within the lungs are significant; they comprise a major portion of cells in both the airways and lung tissue, and they are one of the most important cell types in regulating inflammation progression (both initiation and resolution) [15,43]. In asthma, macrophages are polarized morphologically and functionally to respond to the local cytokine signals, bacteria, allergens, and metabolic factors they detect, resulting in a very dynamic, to a lesser degree, wide-range spectrum of functions that can be classified generally as either a classical M1 or an alternate M2 set of cells [43]. The M1-type macrophages can be induced by IFN-γ, lipopolysaccharides, and pro-inflammatory cytokines, resulting in the activation of both NF-κB and STAT1 signal transduction pathways and the production of TNF-α, IL-1β, IL-6, iNOS, and reactive oxygen species. These mediators collectively amplify airway inflammation, increase airway epithelial cell death from injury, and increase oxidative stress and tissue destruction, thereby increasing the risk of the development of persistent airway hyperresponsiveness and asthma attacks [43].

In contrast to M1 macrophages, M2 macrophage activation is predominantly induced by IL-4 and IL-13 and is associated with processes that lead to tissue repair, remodeling of the extracellular matrix and the production of anti-inflammatory signals. M2 macrophages secrete several mediators, including IL-10, TGF-β, Arg1 and several chemokines (e.g., CCL17 and CCL22), all of which facilitate eosinophilic inflammation, recruitment of Th2 cells and the remodeling of airways [15,43].

Despite M2 macrophages generally being considered responsible for the resolution of inflammation and tissue repair, their function within the airways of asthmatics is significantly more nuanced. Chronic M2 macrophage activation can also lead to airway remodeling in asthma through the release of TGF-ß, growth factors and matrix-modifying enzymes. Overactivity of the M2 pathway may therefore be associated with the pathogenesis of subepithelial fibrosis, goblet cell hyperplasia and mucus hypersecretion, as well as airway smooth muscle remodeling and chronic airflow limitation. M2 macrophages appear to mediate both protective and damaging roles dependent on the inflammatory context and duration of activation [15,20,38].

Therefore, maintenance of a functional equilibrium between pro-inflammatory M1 and pro-repair M2 function is essential for airway immune homeostasis. While the M1/M2 classification provides a useful means of describing general macrophage functions, it is now well established that the regulation of macrophage activation is more complex than the two discrete dichotomous states previously considered and has been redefined as a continuum. Marked heterogeneity of macrophages has been demonstrated in the asthmatic airway, with sub-populations being defined as inflammatory, reparative, metabolic and regulatory. It is likely that M1/M2 is merely a conceptual, and ultimately imprecise, tool and not representative of the authentic phenotype [15,19].

The airway macrophage population is composed of both tissue-resident and monocyte-derived macrophages, which differ both in origin and function. Airway resident alveolar macrophages are responsible for the maintenance of homeostasis by performing immune surveillance, phagocytosing inhaled particles and controlling local immunity. Monocyte-derived macrophages are recruited to the airway during inflammation and are often more potent inflammatory cells, promoting the production of cytokines, tissue damage and airway remodeling. Dysregulation of both these macrophage populations has been implicated in severe asthma and failure of inflammatory resolution [15,19,44].

More recently, progress made with the analysis of single-cell RNA sequencing has improved our knowledge of the macrophage heterogeneity in asthma. Transcriptomic profiles using single-cell RNA sequencing revealed that there exists a multitude of macrophage subsets that are associated with a distinct inflammatory signature, interferon-responsive signature, profibrotic signature, and pro-resolving signature, which were associated with asthmatic airways. This indicates that macrophage function is actually far more complex than the simplistic M1/M2 model and has opened the possibility for more precise targeted therapies against certain macrophage subsets [15,45,46].

In addition to producing cytokines, macrophages have key roles in: (i) presenting antigen to T lymphocytes, (ii) clearing away (i.e., phagocytosis and efferocytosis) dead cells and other cellular debris from tissues that are inflamed, and (iii) feeling “full” after macrophage activation causes them to lose the capacity to clear apoptotic cells (eosinophils) and necrotic cellular debris. Macrophage efferocytosis dysfunction in patients with severe asthma leads to the buildup of apoptotic eosinophils as well as necrotic cellular debris (a source of activators of NF-κB), resulting in continued inflammatory signaling by NF-κB and failed resolution of inflammation. Studies are beginning to demonstrate that macrophages’ metabolism is associated with their polarization states, such that M1 macrophages primarily use glycolysis for their energy requirements while M2 macrophages primarily utilize oxidative phosphorylation and fat oxidation for their energy requirements. These two “metabolic programmers” for M1 and M2 macrophages modulate the extent to which the macrophages respond to pro-resolution mediators, to inflammatory signals, and to remodeling of the affected tissue [9,15,47].

Specialized pro-resolving mediators (SPMs), including lipoxins, resolvins, protectins, and maresins, have many of their therapeutic actions mediated by the reprogramming of macrophages, with SPMs down-regulating the inflammatory responses of M1-like macrophages and up-regulating the efferocytosis and tissue homeostasis restoration functions of M2-like macrophages. Thus, macrophages are key cellular mediators linking SPM therapies to chronic airway inflammation, defective resolution and SPM-directed therapies in asthma [15,48,49,50]. The major macrophage subtypes, their activation markers, and their pathological roles associated with asthma are summarized in Table 1.

### 2.6. Steroid Resistance and Macrophage-Mediated Inflammation

While corticosteroids form the basis for treating inflammation in asthma, many with persistent and difficult-to-control severe asthma demonstrate partial or full resistance to corticosteroids and continue to have persistent airway inflammation and poor control of their asthma [59,60]. Recent studies provide evidence that macrophages are instrumental in the establishment and persistence of steroid-resistant inflammatory processes via aberrant glucocorticoid receptor (GR) signaling; sustained activation of pro-inflammatory transcription factors; and defective resolution mechanisms [43,53].

Airway macrophages in asthma patients that are unresponsive to corticosteroids demonstrate increased regulation of the NF-κB, p38 MAPK and PI3K/Akt pathways, which results in the long-term production of inflammatory mediators such as TNF-α, IL-1β, IL-6, CXCL8 and reactive oxygen species, even when the patient has received corticosteroid treatment [43,61]. Patients who are exposed to high levels of oxidative stress also have reduced histone deacetylase-2 (HDAC2) activity, which is necessary for the glucocorticoid-mediated inhibition of the transcription of inflammatory genes. Reduced HDAC2 in macrophages is associated with ongoing inflammation and reduced sensitivity to steroids in patients with severe asthma [62,63].

An imbalance in macrophage polarization is also associated with resistance to steroid therapy. Continuous inflammatory activation via M1 macrophages leads to pro-inflammatory cytokines and injury to the lungs, while abnormal M2 responses contribute to airway remodeling and fibrosis but do not restore immune stability. Beyond this, inadequate removal of apoptotic cells by macrophages in severe asthma leads to increasing numbers of apoptotic cells and secondary necrosis and thus exacerbates chronic inflammation and perpetuates corticosteroid-resistant diseases. According to some recent investigations, specialized proresolution mediators (SPMs) could assist in combating steroid resistance due to their ability to reprogram macrophages into proresolution phenotypes by enhancing efferocytosis and by modulating pathways involved in NF-κB-mediated inflammation without causing generalized immunosuppression. Modulating macrophage signaling and inflammatory metabolism through the use of SPMs holds promise as part of a therapeutic approach to restoring resolution pathways, improving the treatment response in patients experiencing severe asthma [43,53,61].

### 2.7. Inflammatory Mechanisms Underpinning Non-Type 2 Asthma

Asthma, which is not Type 2, may be further subdivided into types of inflammation (neutrophilic, mixed granulocyte or few granulocytes). These non-Type 2 phenotypes of asthma have been shown to be correlated to Th1 and Th17, these forms of asthma being not related to the Type 2 eosinophilic response. The Type 1/17 phenotypes have been associated with an increase in clinical severity, with sustained inflammation from mediators such as Interferon gamma (IFN-), IL-6 and IL-17, resulting in steroid resistance. Macrophages may be extremely important in non-type 2 asthma due to sustained pro-inflammatory responses (release of cytokines) and generation of free radicals and failed resolution. Persistent neutrophilic inflammation and failure of efferocytosis may result in ongoing damage and remodeling of the airway, leading to poor therapeutic response in patients with no readily available biomarkers and difficulty in managing those patients. Macrophage/microglial function and resolution pathways could be future targets. It may be true that distinct resolution defects are observed in the various asthmatic endotypes. It has been suggested that severe neutrophilic asthma and non-Type 2 phenotypes may exhibit an inability to generate SPMs efficiently and resolve inflammation effectively, but there may be limited clinical data currently supporting these hypotheses. Taken together, these observations seem to support the role of impaired macrophages and failure of SPMs in non-type 2 asthma in exacerbating inflammation and causing steroid resistance [63,64,65].

### 2.8. Defective Inflammatory Resolution in Asthma

Research indicates that asthma is not only a chronic inflammatory disorder but also a disease with abnormalities in the resolution of inflammation [66,67]. Normally, following acute or short-term inflammation, there is a period during which inflammation resolves. This process includes stopping the activation of cells that cause inflammation and clearing the remains of those cells via macrophages. Also, during this period, restoration of barrier integrity occurs between the epithelial cells of the respiratory and digestive systems and the tissues within those systems, thus allowing for the repair of damaged tissues associated with the original episode of inflammation. When the coordination of resolution pathways becomes defective, as seen with ongoing episodes of eosinophilic–neutrophilic inflammation, ongoing cytokine production, and continued injury to the airways, then the ultimate damage to the airways can become chronic [66].

Deficient resolution of asthma has been associated with a decrease in biosynthesis and activity of specialized pro-resolving mediators (SPMs), including lipoxins, resolvins, protectins, and maresins. Several studies have shown that these endogenous mediators were lower in individuals with severe and uncontrolled asthma, and the decreased levels could be due to altered lipoxygenase activity, oxidative degradation of the SPMs, dysregulated fatty acid metabolism, and impaired receptor signaling [66,68]. The persistent absence of SPMs leads to the continued activation of pro-inflammatory pathways (NF-κB, MAPK, STAT6 and PI3K/Akt), resulting in the ongoing production of IL-4, IL-5, IL-13, TNF-α and other inflammatory mediators that maintain airway inflammation and tissue remodeling.

Macrophage dysfunction is key to this resolution process failing. Macrophage dysfunction in asthma is not due to an abnormal distribution of M1 and M2 macrophage subsets but involves altering macrophage plasticity, activation and pro-resolving activities. Macrophage polarization and efferocytosis are ineffective in severe asthmatic individuals, resulting in inefficient removal of apoptotic eosinophils and inflammatory debris and continuing chronic inflammation and oxidative stress [51,67]. Increased levels of reactive oxygen species negatively impact SPM biosynthesis and glucocorticoid receptor signaling to further contribute to corticosteroid resistance and chronic airway inflammation. As a result, ongoing inflammation creates an environment for airway remodeling that is characterized by fibrosis, goblet cell hyperplasia, smooth muscle hypertrophy, and deposition of extracellular matrix. The data presented in this article support the idea that restoring endogenous resolution pathways through reprogramming macrophages and using SPM-based therapies may provide an effective means of controlling chronic airway inflammation and restoring immune homeostasis in asthmatics [66,68].

## 3. Specialized Pro-Resolving Lipid Mediators (SPMs)

The endogenous lipid molecules called SPMs have a crucial role in managing the end of the inflammatory process, the repair of damaged tissue, and the return of an individual to homeostasis while still providing effective immune defense. In contrast with typical non-steroidal anti-inflammatory drugs (NSAIDs), which suppress many aspects of immune function broadly, SPM actively terminate ongoing inflammation due to their ability to modulate a variety of cellular responses to avoid the development of chronic inflammation and resultant tissue injury [69,70]. These mediators are biosynthesized from essential polyunsaturated fatty acids (PUFAs), primarily omega-3 and omega-6 fatty acids, through tightly regulated enzymatic pathways involving lipoxygenases and cyclooxygenases [70]. Timely production of SPMs allows clearance of apoptotic cells (efferocytosis) by macrophages and restoration of tissue integrity, and thereby avoids pathologies associated with unresolved inflammation [69,70].

**Figure 3:**
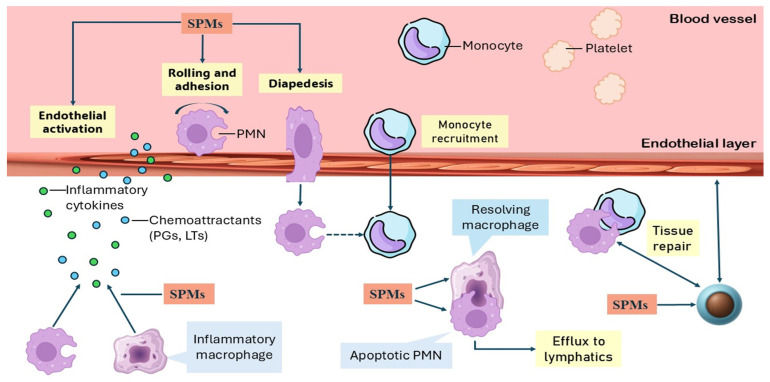
Role of Specialized Pro-Resolving Lipid Mediators (SPMs) in inflammation resolution: This schematic illustrates the multifaceted roles of SPMs in orchestrating the resolution phase of inflammation. SPMs act on endothelial cells to inhibit activation, reducing the adhesion and rolling of polymorphonuclear leukocytes (PMNs) and their diapedesis across the endothelial layer into inflamed tissues. They modulate the recruitment and phenotype of monocytes, promoting their differentiation into resolving macrophages. Resolving macrophages facilitate the clearance of apoptotic PMNs through enhanced efferocytosis and support the efflux of cellular debris to the lymphatics. Additionally, SPMs stimulate tissue repair mechanisms by acting on structural cells. This coordinated immune regulation curtails ongoing inflammation, promotes clearance of inflammatory cells, and restores tissue homeostasis, underscoring SPMs as critical mediators of natural inflammation resolution.

There have been new studies showing how SPMs can be beneficial in treating certain inflammatory conditions. For example, some researchers have found that SPMs can help respiratory diseases (such as Asthma and COPD) reduce mucus hypersecretion and bronchial hyperreactivity, and maintain an appropriate immune balance, thereby providing protective effects against airway remodeling and excessive inflammation [69,70,71]. Moreover, omega-3 polyunsaturated fatty acid-derived specialized pro-resolving mediators have been documented to improve the functioning of the gastrointestinal barrier, modify immune reaction and restore balance of the gut microbiota in patients with inflammatory bowel disease. These observations demonstrate some of the broader immunomodulatory properties of these lipid mediators [72]. Figure 3 highlights the major role of SPM’s in coordinating the processes that limit the number of leukocytes within ingested dead cells and the restoration of tissue homeostasis [70,73]. These accumulating data highlight the important role of SPMs in the resolution of physiological inflammation and position them as promising therapeutic agents in inflammatory and autoimmune disorders [69,70].

### 3.1. SPMs Versus Classical Anti-Inflammatory Drugs

Both SPMs and conventional anti-inflammatory drugs target the resolution of inflammation, but their mechanisms and biological outcomes are fundamentally different [74]. Classical agents such as corticosteroids and non-steroidal anti-inflammatory drugs (NSAIDs) mainly work by inhibiting the synthesis of pro-inflammatory mediators (e.g., by blocking COX or NF-κB pathways) and thus suppressing cytokine release and leukocyte activation [75]. The use of anti-inflammatory medications is effective in reducing acute inflammation but can hinder the functional ability of hosts to defend themselves from pathogens, prolong the repair process, and produce systemic adverse effects such as suppression of the immune system, gastrointestinal toxicity and dysregulation of metabolism. On the contrary, SPMs do not inhibit inflammation but play a role in coordinating its resolution while enhancing the clearance by macrophages of destroyed cells and cellular debris, enhancing the repair of epithelial cells, as well as modulating the immune system to return to a steady state [74,75]. Importantly, SPMs maintain antimicrobial immunity and tissue integrity, providing a balanced immunoregulatory approach without the collateral effects seen with chronic corticosteroid or NSAID use [75]. This mechanistic difference highlights the emerging paradigm of resolution pharmacology—a therapeutic approach aimed at restoring physiological healing, rather than blocking inflammatory signals.

### 3.2. Classification of Specialized Pro-Resolving Lipid Mediators (SPMs)

**Figure 4:**
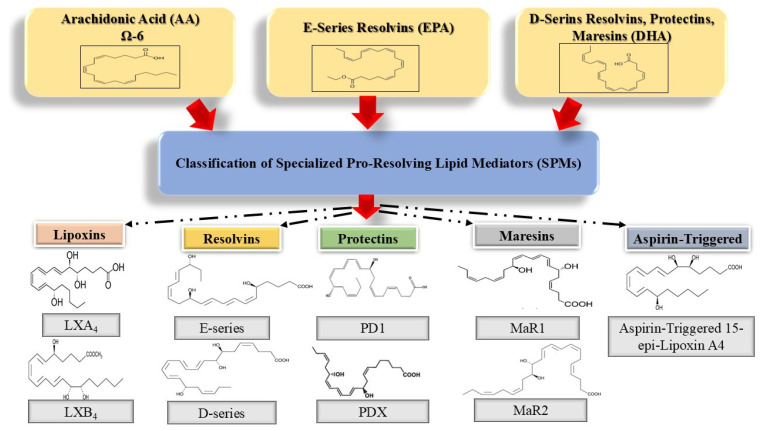
Classification and biosynthetic origins of specialized pro-resolving lipid mediators (SPMs). This schematic shows how specialized pro-resolving lipid mediators (SPMs) are classified based on their polyunsaturated fatty acid precursors and the structural family they belong to. The two primary polyunsaturated fatty acids that are used by SPMs as starting materials are arachidonic acid (AA, ω-6), which is converted to lipoxins (LXA_4_, LXB_4_) and aspirin-triggered 15-epi-lipoxins by lipoxygenase or acetylated cyclooxygenase-2 pathways; and eicosapentaenoic acid (EPA, ω-3), which is converted to E-series resolvins by means of enzymes. The second class of polyunsaturated fatty acids used to make SPMs is docosahexaenoic acid (DHA, ω-3), which is used to make D-series resolvins, protectins (PD1, PDX), and maresins (MaR1, MaR2). SPMs are created through the coordinated actions of 5-, 12- and 15-lipoxygenases and, in the presence of aspirin, by acetylated COX-2, resulting in SPMs in their epimeric forms, possessing increased metabolic stability relative to non-epimeric SPMs. Each of the SPM families is capable of acting as an endogenous signal for resolution of inflammation, promoting the reprogramming of macrophages, enhancing efferocytosis and restoring tissue homeostasis during inflammatory diseases like asthma.

Specialized pro-resolving lipid mediators (SPMs) make up a superfamily of bioactive lipids. These lipids are produced through enzymes and play a key role in controlling the resolution of inflammation [76,77]. SPMs can stop the inflammatory process by reprogramming cells and restoring tissue to a state of homeostasis. They also allow for rapid and improved repair of tissues without compromising the immune function of the body [75,78]. SPM are formed by morphological precursors known as polyunsaturated fatty acids (PUFA), which exhibit a diverse array of structural types, the primary types of which being arachidonic acid (AA; 20:4, ω-6), eicosapentaenoic acid (EPA; 20:5, ω-3) or docosahexanoic acid (DHA; 22:6, ω-3) utilizing the concerted efforts of lipoxygenases 5-lipoxygenase, 12-lipoxygenase, and 15-lipoxygenase and cyclooxygenases (COX-1/2) to synthesize SPM from these PUFA [76,79,80]. The chemical structure and biogenesis of SPMs can be grouped into four families of molecules: lipoxins, resolvins (E-series and D-series), protectins and maresins. There are also aspirin-induced, epimerized derivatives that could be considered another form of SPM, but they fall under two categories for pharmacological classification [76,77,81]. In Figure 4 (Classification and biosynthetic origin of specialized pro-resolving lipid mediators (SPMs)), the structural classifications of these mediators and the pathways for their biosynthetic production are presented [76,79]. All of these mediators, despite their differing macro-structure, are interacting with GPCRs (G protein-linked receptors), yielding receptor-mediated interactions that decrease the pro-inflammatory pathways of NF-κB and MAPKs, while simultaneously enhancing the macrophage’s efferocytosis, epithelial repair and tissue regeneration [75,78]. The subsequent sections of this document will provide an explanation of each of the subclasses of SPMs. These explanations will include information on the biochemical origin of SPM subclasses, their receptor interactions, molecular mechanisms, and functional roles, particularly regarding the roles of SPMs in asthma and chronic airway inflammation [70,78].

#### 3.2.1. Lipoxins (LXA_4_, LXB_4_)

Lipoxins were the first SPMs discovered and are still among the most thoroughly investigated SPMs involved in asthma. The biological activity of lipoxins appears to occur via activation of the ALX/FPR2 receptor found on neutrophils, macrophages, and epithelial cells [75,78]. Lipoxins have been shown to downregulate neutrophils and neutrophil infiltration, decrease chemokine production and increase macrophage efferocytosis and downregulate NF-B and MAPK signaling to suppress pro-inflammatory and increase anti-inflammatory mediator production [70,78]. It has been demonstrated that in vivo, LXA suppresses eosinophilic inflammation, hyperresponsiveness and inflammatory cytokine release and upregulates macrophage-dependent pathways of resolution [70]. Clinical studies in humans also demonstrate low LXA levels in bronchoalveolar lavage fluid and other samples from severe asthmatics, indicating that a lipoxin deficit contributes to a failure of inflammatory resolution [71]. In summary, lipoxins are important endogenous regulators of inflammation resolution and one of the most characterized SPM subsets.

#### 3.2.2. Resolvins (E-Series and D-Series)

Among specialized pro-resolving mediators, resolvins are the best characterized and possess profound anti-inflammatory resolution effects. Biology of resolution mediators, including resolvins, is controlled mainly by GPCRs such as ChemR23, GPR32 and GPR18 receptors. Activation of ChemR23, GPR32 and GPR18 suppresses pro-inflammatory mediators, increases efferocytosis and shifts macrophages to pro-resolution phenotypes [75,78]. In vitro and vivo experiments showed that RvD1 suppressed Th2 cytokine synthesis, eased eosinophilic inflammation and enhanced tissue repair, returning to the immune homeostasis [69,70]. The same effects, decreased airway hyperresponsiveness and inflammatory mediators via inhibiting NF-B dependent signaling, were demonstrated when the mouse model was administered with RvD1 and RvE1. Moreover, it had been found that the lack of RvD1 was correlated to the increased intensity of asthma and failure of inflammatory resolution in clinical studies [75,78]. To sum up, Resolvins are one of the most characteristic members of the SPM family, which could potentially treat the chronic airway inflammatory disease.

#### 3.2.3. Protectins (PD1, PDX)

Protectins, especially Protectin D1 (PD1), are DPA-derived specialized pro-resolving mediators that show powerful anti-inflammatory and tissue-protective functions [76,77]. PD1 triggers receptor-mediated pathways to restrict leucocyte infiltration, promote macrophage efferocytosis and maintain the integrity of the epithelial barrier and stimulate tissue healing [69,70,78]. In addition, protectins inhibit pro-inflammatory signaling and promote the expression of tissue homeostasis-related and resolution-specific genes [78]. PD1 attenuated recruitment of inflammatory cells and induced an increase in IL-10, accelerated the clearance of apoptotic cells and promoted the resolution of epithelial repair [70] in animal models of lung injury and airway inflammation. In animal models of airway diseases, it has been found that PD1 protects the airways from the damage caused by inflammation. In contrast to the relatively large amount of data supporting lipoxins and resolvins in the clinics, the use of protectins in managing chronic airway inflammatory diseases, including asthma, is yet at the nascent stages, but its strong effects in restoring tissue homeostasis and resolving inflammation cannot be overlooked [70].

#### 3.2.4. Maresins (MaR1, MaR2)

Maresins (MaR1 and MaR2) are particular subsets of pro-resolving mediators produced mainly by macrophages and implicated in the resolution of inflammation and tissue repair [82,83]. Maresins can stimulate macrophage efferocytosis, suppress pro-inflammatory mediators’ production, increase anti-inflammatory cytokines’ production and promote restoration of tissue homeostasis [82,83,84]. Pre-clinical data revealed that MaR1 attenuated airway inflammation, prevented collagen deposition and airway remodeling, restored epithelial barrier integrity and enhanced lung function in a mouse model of asthma [85,86]. Maresins can also augment macrophage-mediated removal of apoptotic cells and promote resolution toward a pro-resolving environment. Recent reports showed evidence of reduced maresins levels associated with prolonged inflammation and defective tissue repair in severe asthma. Hence, maresins are considered suitable therapeutic agents due to their beneficial effects in both reducing inflammation and promoting tissue regeneration/repair [82,83,84,85,86].

#### 3.2.5. Aspirin-Triggered SPMs (AT-SPMs)

Aspirin-triggered specialized pro-resolving mediators (AT-SPMs), such as AT-LXA and AT-RvD1, are epimeric variants of SPMs that are derived by aspirin-mediated modulation of cyclooxygenase-2 [87,88]. Compared with their native analogs, AT-SPMs typically display a more resistant metabolic profile while retaining similar potency and pro-resolving activity. Existing literature supports that the primary signaling pathway for AT-SPMs is via the ALX/FPR2 receptor, one of the most well-characterized SPM receptors. The signaling cascade initiated by ALX/FPR2 activation leads to enhanced macrophage efferocytosis, inhibition of excessive neutrophil recruitment and inflammatory cytokine production, as well as recovery of tissue homeostasis [87,89]. In experimental models, AT-LXA and AT-RvD1 attenuated both airway inflammation and fibrosis as well as inflammatory signaling [90,91]. Therefore, they may be therapeutic candidates for chronic inflammatory diseases such as asthma. While preclinical results for AT-SPMs in asthma appear promising, limited clinical data are available. Further investigation is warranted to elucidate their efficacy and delivery strategies, as well as the safety profile for the management of severe and steroid-resistant asthma.

In order to effectively distinguish preclinical results from clinical observations, the available data for each SPM subclass are summarized in Table 2. The table shows experimental species, model systems used, biological samples studied, principal findings, and any clinical findings or inconsistencies and limitations reported between the various studies.

#### 3.2.6. Critical Appraisal of SPM Families in Asthma

While all SPM families possess pro-resolving effects, the degree of evidence supporting the role of each family in asthma varies. The lipoxins and resolvins have been shown to possess the strongest experimental and clinical evidence of all SPM subclasses to date. A decrease in LXA, RvD1 and RvE1 levels has been observed in asthmatic patients (especially with severe disease), and Administration of these mediators suppresses airway inflammation, eosinophilia, and airway hyperresponsiveness in a variety of preclinical asthma models. Lipoxins and resolvins are the best-characterized SPM families in asthma at present. ALX/FPR2 is the best characterized and most validated receptor in SPM signaling in asthma to date, mediating many of the lipoxin and ATL-SPM activities and repeatedly identified to be involved in macrophage reeducation, enhancing efferocytosis and inhibiting airway inflammation. ChemR23, GPR32 and GPR18 were reported to be receptors for particular Rv, with a limited level of experimental evidence compared with ALX/FPR2. However, there remain shortcomings and limitations. A few clinical studies have been conducted to investigate the role of the protectins, maresins and ATLs. The evidence for the involvement of the protectins, maresins and ATLs in asthma largely derives from in vitro and preclinical model systems. Little is known about the exact involvement of particular SPMs in each asthma endotype, especially non-type 2 asthma and neutrophilic asthma. Inconsistent results for SPM measurement are attributable to variation in methodology, patients and disease severity. Overall, at the present time, the lipoxins and resolvins provide the strongest targets for therapy in asthma, but additional clinical studies are needed to ascertain the clinical utility of other SPM subclasses in this disease. Their utility in steroid-resistant and non-type 2 asthma is currently unknown [10,15,49,96].

## 4. Molecular Pharmacology of SPM Receptors

### 4.1. Receptor Structure and Ligand Binding

SPMs have a function by way of attaching themselves to particular GPCR subtype(s), specifically the members of Class A (or rhodopsin-like), which is the largest, evolutionarily conserved sub-group within all GPCRs. All Class A GPCRs have been established as also having 7-transmembrane alpha helical domains with an intracellular C-terminus and an extracellular N-terminus. These conserved 7-TM characteristics of Class A GPCRs allow for the coupling of heterotrimeric G-proteins and the initiation of intracellular signaling cascades through agonist-driven conformational changes occurring in the transmembrane bundle [97].

The various types of GPCRs in the Class A Superfamily of the GPCR (ALX/FPR2, ChemR23, GPR32, GPR18 and GPR37) pass on lipid signals from the outside to the inside of a cell at the level of a lipid ligand with a nanomolar affinity, thereby leading to a chemical change within the cell biochemically, caused by a receptor [98]. Binding of SPM to GPCRs induces conformational changes within the transmembrane helices and intracellular loops to alter the orientation of these regions, which then allows for GDP-to-GTP exchange on cognate Gα subunits and activates downstream effectors such as the phosphoinositide 3-kinase (PI3K)/Akt pathway, mitogen-activated protein kinase (MAPK) pathways, and β-arrestin-dependent mechanisms. By applying high-resolution structural methods, it has been demonstrated that Class A GPCR ligand binding sites are comprised predominantly of residues from transmembrane helices. By binding an agonist, the activated state of the receptor is stabilized through movement of TM6 outward and rearrangement of its conserved “microswitch” motifs, allowing for efficient coupling of proteins to GPCR and transmission of the signal [97].

Several groups using molecular docking and molecular dynamics simulation approaches have identified possible binding modes of resolvins, such as RvD1, in ALX/FPR2. These results must be confirmed experimentally and be viewed as corroborating, rather than establishing, the receptor-ligand interactions [99].

### 4.2. Signal Transduction and Biased Agonism

**Figure 5:**
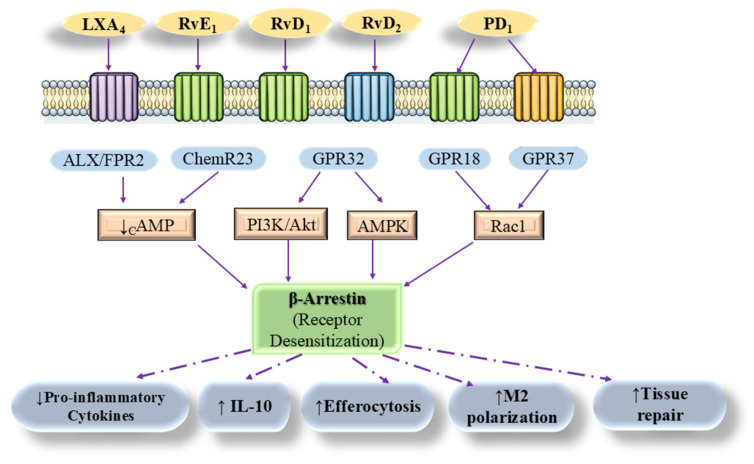
Receptor-mediated signaling mechanisms and pro-resolving functional outcomes of specialized pro-resolving mediators. Lipoxin A_4_ (LXA_4_), resolvin E1 (RvE1), resolvin D1 (RvD1), resolvin D2 (RvD2), and protectin D1 (PD1) are important Major SPMs that signal through specific Class A GPCRs (ALX/FPR2, ChemR23, GPR32, GPR18, GPR37). When bound to their ligands, the Gαi-dependent signaling inhibits cAMP production and activates PI3K/Akt, AMPK, and Rac1 pathways and causes the recruitment of β-arrestin. By coordinating these signaling cascades, they inhibit NF-κB-mediated transcription of pro-inflammatory genes, enhance IL-10 production, promote efferocytosis, M2 macrophage polarization, and tissue repair, and promote resolution of inflammation.

Specialized pro-resolving mediator (SPM)-induced intracellular signaling transduction processes, initiated by their G-protein-coupled receptors (GPCRs), are primarily linked to Gαi/o proteins that inhibit adenylyl cyclase activity (decreasing cAMP production) and thus also enable the activation of pro-resolution signaling pathways such as PI3K/Akt and MAPK. Initial research has indicated that several SPM receptors (e.g., ALX/FPR2, ChemR23) are potential candidates for showing characteristics of biased agonism in which ligands have preferential effects on downstream signaling pathways. However, the degree of biased signaling across the entire family of SPM receptors and the biological relevance are not fully elucidated. In addition to being able to selectively signal through functional selectivity, these receptors are sufficiently functionally selective as to permit the biased signaling of ligands toward either anti-inflammatory (e.g., increased efferocytosis, decreased NF-κB) or pro-resolution signaling outcomes rather than pro-inflammatory signaling pathways, thus providing for increased therapeutic specificity and reduced unintended consequences [76]. The discovery of positive allosteric modulators for various GPCRs, such as the prostaglandin E_2_ Receptor EP4, through the use of these small-molecule ligands underlines the ways these molecules can convert GPCR coupling towards pro-resolution phenotypes in macrophages and provide for enhanced G_S_-mediated cAMP signaling. The existence of these biased signaling profiles represents an example of the highly complex molecular pharmacology of SPM-GPCR systems, as well as their potential to be used as drugs that are pathway-selective in treating chronic airway inflammation. Table 3 provides summaries of the various pharmacological and signaling characteristics for these compounds, and Figure 5 illustrates receptor-specific signaling pathways along with downstream functional effects associated with each of the three major classes of SPMs [100].

### 4.3. Biochemical Stability and Metabolism

Pharmacokinetics (PK) is the study of the ways in which a drug interacts with the body in terms of the way that a drug is absorbed, distributed, metabolized, and eliminated from the body (ADME or ADME). A key limitation of SPMs is that they have a very short biological half-life due to their relative instability and rapid metabolism by the body. Indeed, the native form of SPMs are most often found in very low concentrations and only for short periods of time within inflamed tissue and body fluids due to the fact that they are susceptible to enzymatic degradation through the actions of various metabolic pathways, including oxidations performed by lipoxygenases, dehydrogenations performed by prostaglandin dehydrogenases, and isoforms of cytochrome P450; thus, SPMs will often only be seen with transient biological activity [10,49].

There are many different groups of SPMs. One area of interest is how long each of the distinct types of SPMs remains in the bloodstream after their formation. Based on available pharmacokinetic studies, native lipoxins show extremely short half-lives in plasma, with values varying between experiments depending on the methods used. Second, resolvins also have very short half-lives (i.e., a few minutes), depending on the experimental conditions and whether they are E- or D-series resolvins. Third, protectins and maresins are slightly more resistant to metabolism than lipoxins and resolvins since protectins and maresins can remain intact for a short period of time, approximately minutes, during inflammatory processes. In contrast, aspirin-triggered SPMs (i.e., AT-SPMs) such as aspirin-triggered lipoxin A_4_ (AT-LXA_4_) and aspirin-triggered resolvin D1 (AT-RvD1) are more stable than their respective native SPMs because of their epimeric chemical structure, which allows them to resist the rapid metabolism typically associated with other SPMs [76,107]. The approximate half-lives in plasma and biochemical stability of the major classes of SPMs are summarized in Table 4.

SPM biosynthesis has been found to be reduced as a result of oxidative stress and chronic inflammation associated with severe asthma and other diseases; therefore, the pro-resolving ability of SPM would also be reduced due to the increased degradation of the compounds. The presence of elevated ROS, lipid peroxides, and altered fatty-acid metabolism has all been implicated in the reduced production of SPM. Because of these pharmacokinetic (i.e., pertaining to how drugs are absorbed, distributed, metabolized, and eliminated), and metabolic limitations (i.e., pertaining to how a drug is metabolized by the body), there is considerable interest in the development of synthetic SPM analogs, nanoformulations (containing nanoparticles), liposomal delivery systems (i.e., drug-delivery systems using lipids), and receptor-targeted agonists designed to enhance the bioavailability of SPM, extend the duration of SPM in the tissues, and enhance the therapeutic benefits of SPM, while continuing to utilize the endogenous resolution pathways of the host [108,109,110,111].

**Table 4:** Approximate Plasma Half-life and Relative Biochemical Stability of Major Specialized Pro-Resolving Mediator (SPM) Subclasses.

SPM Subclass	Approximate Plasma Half-Life (t½)	Relative Stability	References
Lipoxins	Seconds to a few minutes	Very low	[76,79,111]
Resolvins	Few minutes	Low	[76,79,111]
Protectins	Several minutes	Moderate	[76,79]
Maresins	Several minutes	Moderate	[76,79]
Aspirin-triggered SPMs	Longer than native SPMs	Relatively improved	[76,79,111]

## 5. Molecular Mechanisms of Macrophage Reprogramming

**Figure 6:**
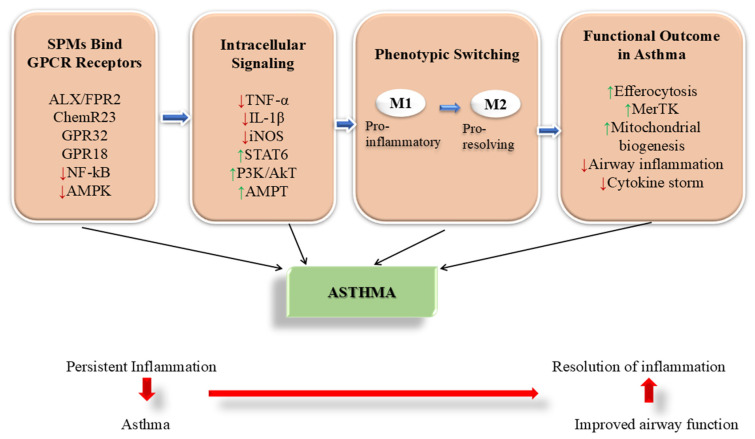
Integrated mechanism of SPM-mediated resolution pathways in asthma. Specialized pro-resolving mediators (SPMs) initiate intracellular signaling cascades when they bind to G protein-coupled receptors (GPCRs) such as ALX/FPR2, ChemR23, GPR32, and GPR18. Activation of these receptors suppresses pro-inflammatory mediators (TNF-α, IL-1β, iNOS) and nuclear factor-kappa B (NF-κB) activity and enhances the activities of pro-resolving pathways (i.e., signal transducer and activator of transcription (STAT) 6, phosphoinositide-3-kinase (PI3K/Akt), and AMP-activated protein kinase (AMPK)). These signaling pathways promote phenotypic switching of macrophages from the classically activated M1 (pro-inflammatory) phenotype to the alternatively activated M2 (pro-resolving) phenotype. These functional outcomes include improved efferocytosis, increased expression of MerTK, improved mitochondrial biogenesis, and decreased inflammation and cytokine production in the airways. Collectively, these mechanisms provide a shift from persistent airway inflammation to active resolution, assisting in the reestablishment of homeostasis in the airway and improving lung function in patients with asthma.

Macrophage plasticity is a significant factor in regulating and influencing chronic inflammation in long-standing airway diseases such as asthma. SPMs (specialized pro-resolving mediators) utilize a large array of mechanisms to modulate macrophage reprogramming, including receptor-mediated signaling (primarily via class A GPCRs), regulating transcription, regulating metabolism and enhancing efferocytosis. SPMs, through the stimulation of class A GPCRs, inhibit M1-related pathways while promoting the development of an M2 macrophage phenotype, leading to a restored immunologic balance as well as improved tissue repair. Together, these processes enable resolution and restore chronic homeostasis in the airways of individuals with asthma Figure 6 [10,43,70,75,112].

### 5.1. Receptor-Mediated Initiation of Phenotypic Switching

**Figure 7:**
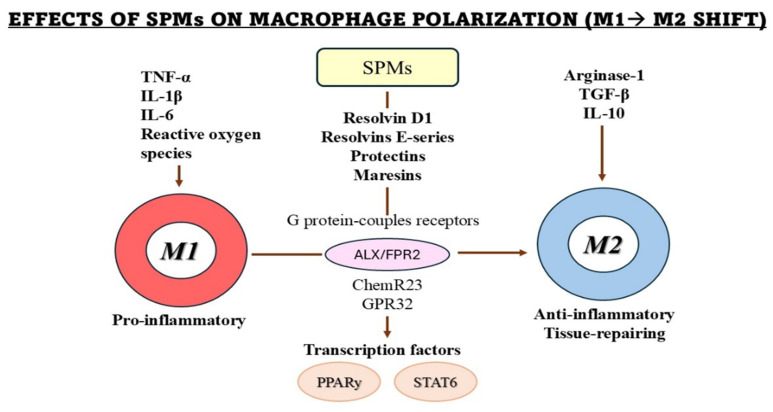
SPM-Mediated Resolution Network in Asthma: Such molecular processes lead to the regulated shift from pro-inflammatory M1- to pro-resolving M2-like macrophages and, in turn, improve efferocytosis and tissue repair. Nevertheless, chronic or over-polarized M2 may promote detrimental tissue remodeling and fibrosis, showing that well-balanced macrophage reprogramming is necessary for proper inflammation resolution.

Specialized pro-resolution mediators (SPM) reprogram macrophages by engaging the activity of class A G-protein-coupled receptors (GPCRs; ALX/FPR2, ChemR23, GPR32, and GPR18) through the same agonists that activate these receptors [112]. More than half of the class A GPCRs, including these identified SPM GPCRs, couple to Gαi proteins and activate downstream signaling pathways, including phosphoinositide-3 kinase (PI3K), Akt, and AMPK activation, while inhibiting nuclear factor kappa B (NF-κB) activity. Proinflammation during an inflammatory response drives M1 polarization via the activation of the transcription factor Signal transducer and activator of transcription 1 (STAT1), by the transcription factor Interferon regulatory factor 5 (IRF5) or by NF-κB activation increasing production of pro-inflammatory mediators including Tumor Necrosis Factor alpha (TNF-α), Interleukin 1 beta (IL-1β), Interleukin 6 (IL-6) and Inducible nitric oxide synthase (iNOS) [75,112]. Signal by SPM inhibits the M1 polarization pathway through inhibiting phosphorylation of IkappaB Kinase (IKK), which stops IκB degradation, prevents translocation of NFκB p65 subunit into the nucleus, inhibits the activation of STAT1, and decreases IRF5 expression [112]. Concurrently, SPMs also activate transcriptional activation and nuclear translocation of STAT6 and transcriptional activation of PPAR, respectively, and both are indispensable to activate the M2 polarization. Thus, it acts as a molecular switch to promote alternative macrophage by synergistic transcriptional repression of transcriptional drivers that activate pro-inflammatory transcriptional pathways and transcriptional activation of transcriptional regulators that activate anti-inflammatory or pro-resolution macrophage activation pathways. Suppression of pro-inflammatory transcriptional drivers and transcriptional activation of pro-resolving transcriptional regulators constitute the molecular switch favoring alternative macrophage activation (Figure 7). Though the SPM-driven M2 polarization is generally thought to be beneficial in terms of inflammation resolution and tissue restoration, macrophages exist along a continuum rather than distinct M1 and M2. It has recently been observed that M2-like activation beyond its necessary limit can cause fibrotic remodeling of the injured tissue due to increased production of TGF and ECMs. Therefore, it is supposed that SPM-mediated changes in macrophage phenotype do not necessarily represent a stable phenotypic change, but rather acontextual control over reprogramming [75,81,112].

### 5.2. Transcriptional and Epigenetic Reprogramming

Epigenetic changes (via chromatin) and intracellular signaling pathways are both expected to have an impact on macrophage polarization. Expression of genes in macrophages will be subject to modulation through the actions of SPM on whether or not certain genes will be expressed. Thus, SPMs are expected to alter the transcriptional regulation of genes involved in inflammation, such as TNF- and IL-1, by changing either histone acetylation or HDAC activity at promoter regions and open chromatin at genes involved in the resolution response, such as IL-10 and Arg1. In addition, many miRNAs are also expected to be regulated through alteration by SPM, thereby influencing a multitude of post-transcriptional networks through induction of anti-inflammatory miRNAs (e.g., miR-146b) and inhibition of pro-inflammatory miRNAs. These alterations will result in a functional shift from a pro-inflammatory phenotype (‘cytokine-producing’, ‘glycolytic’) to a reparative phenotype. The reprogramming of macrophages via epigenetic means, such as modification of chromatin remodeling and histone methylation state to enable/disable genes involved in inflammation and the resolution response, is becoming more established. These changes also provide a mechanism to maintain observed phenotypes long term and may provide therapeutic targets for regaining immune homeostasis in chronic airway disease [10,15,113].

### 5.3. Metabolic Rewiring of Macrophages

Reprogrammed metabolic characteristics of macrophage polarization are referred to as metabolic reprogramming of macrophages. M1 macrophages (activated by pro-inflammatory cytokines) are thought to be larger, more glycolytic (anisotropic), and use aerobic glycolysis to produce ATP in a way similar to that seen with the Warburg effect. They are also characterized by high levels of HIF-1α and a shrunken TCA cycle pathway. M2 macrophages (activated by anti-inflammatory cytokines) preferentially derive their energy to produce ATP from OXPHOS and fatty acid oxidation (fa). SPM receptor-mediated lipid activators/inhibitors are, at least partially, responsible for mediating this transition in macrophage metabolism that occurs during phagocytosis/inflammation via: (i) activating AMPK, (ii) upregulating CPT1A expression for FAO, (iii) promoting mitochondrial biogenesis, (iv) downregulating stabilization of HIF-1α, (v) restoring TCA cycle integrity. The coordination of the two signaling pathways, Akt and AMPK, helps to inhibit glycolytic enzyme expression while promoting respiration (mitochondrial) through enhancing mitochondrial respiratory function, which contributes to the support of an anti-inflammatory gene expression program and increased intracellular longevity. Recent works indicate that metabolic status is directly coupled with epigenetic regulation, in which metabolic intermediaries can dictate histone modifications and the transcriptional programs controlling macrophage responses [75,109,112,114,115].

### 5.4. Macrophage Heterogeneity Revealed by Single-Cell Transcriptomics

The established view of macrophages with distinct M1 and M2 polarity is now an inadequate simplification of macrophage biology; in vivo, macrophages exist on a spectrum of activation states which is dependent on tissue microenvironment, ontogeny and disease state. Particularly, single-cell transcriptomics has been instrumental in elucidating this complexity and allows the definition of distinct clusters of macrophages based upon unbiased gene expression profiles, without the need for the definition of surface markers or canonical polarizing states. In the setting of chronic airway inflammation, these techniques have shown macrophage populations to not only be heterogeneous but to be constantly reprogrammed throughout resolution and progression of disease [116,117,118].

In the lung, tissue-resident alveolar macrophages are key for homeostasis, surfactant clearance and maintenance of a low-inflammatory alveolar environment. They ‘watch over’ this environment, inhibiting unnecessary inflammation, but ensuring epithelial integrity is maintained, as well as local immune balance. By comparison, these resident alveolar macrophages are contrasted with monocyte-derived infiltrating inflammatory macrophages, recruited upon an inflammatory stimulus, to enhance cytokine amplification, recruit other leukocytes and promote prolonged tissue injury in chronic airway disease. Transcriptional analysis, performed in single-cell analyses, has differentiated infiltrating inflammatory macrophages from resident alveolar macrophages [116,117,119,120].

Critically, pro-resolving macrophages expressing genes involved in efferocytosis, lipid metabolism and tissue repair have also been defined through scRNA-seq. It is proposed that such macrophages remove apoptotic cells and promote tissue integrity during the repair stage and resolution of inflammation. The identification of pro-fibrotic/remodeling macrophages expressing TGF-, COL1A1, matrix metalloproteinases, galectin-3 and osteopontin/SPP1 through single cell analysis demonstrates that this population actively contributes to extracellular matrix remodeling and airway fibrosis. Such macrophages may be particularly involved in the pathogenesis of chronic lung diseases and contribute to the structural remodeling and ensuing functional decline [116,119,121,122,123].

The therapeutic potential of SPMs could be profound. Instead of broadly inhibiting macrophage function, SPMs may be targeted to specifically reprogram inflammatory macrophage subsets to pro-resolving phenotypes in which pathways relevant to efferocytosis and lipid handling are enriched. SPMs may be able to bring about the recovery of immune homeostasis while leaving the protective functions of resident macrophages intact. The macrophage heterogeneity as measured by single-cell resolution, therefore, might be a paradigm for SPM-mediated treatments tailored to asthma and other chronic inflammatory airway diseases [117,118,119,120,122,123].

### 5.5. Enhancement of Efferocytosis and Cytoskeletal Remodeling

One important feature of macrophages that helps to resolve inflammation is that they have an enhanced ability to clear away dead and dying cells (efferocytosis) from the inflamed tissue. Specialized pro-resolving mediators (SPMs) can enhance efferocytosis through a number of synergistic mechanisms, such as activating the MerTK receptor and the Rac1 GTPase, promoting cytoskeletal rearrangement, and upregulating scavenger receptors (e.g., CD206 and CD163). By acting through combined activation of PI3K/Akt signaling and Rac1, SPMs are able to regulate actin polymerization and phagosome formation and thereby facilitate the efficient endocytosis of apoptotic cells. The increased efferocytosis resulting from the action of SPMs prevents secondary necrosis and subsequent release of pro-inflammatory intracellular contents, thereby preventing further downstream inflammatory signaling and facilitating resolution of inflammation [48,75,112,124,125].

### 5.6. Integration of Signaling Networks

Whether a macrophage is programmed to be inflammatory or pro-resolution is ultimately dictated by the interplay of the various signaling networks with each other. The SPMs can help to coordinate these various signaling networks by inhibiting the activation of pro-inflammatory signaling networks and activating the pathways for repair. NF-κB signaling is related to the transcription of pro-inflammatory cytokines, so that when NF-κB is not activated, there will be less M1 inflammation. By the same token, by increasing STAT6 and PPARγ signaling, transcription of the genes related to M2 macrophage polarization will increase. Furthermore, activation of AMPK links the energy status of a cell with the stabilization of the macrophage pro-resolving phenotype and is a source of metabolic support for alternate activation of macrophages. Finally, β-arrestin signaling is responsible for the desensitization of receptors and the tune-down of the downstream signaling of these receptors. Therefore, collectively, SPMs modulate and regulate the above signaling networks such that the immune response can be recalibrated in a controlled manner in order to resolve the inflammatory process without causing any generalized immunosuppression [75,112,126].

It should be highlighted that macrophage repolarization from M1 to M2 status should not be perceived as a one-way journey. There is accumulating evidence that suggests that macrophage states lie on a continuum and are dynamically reversible in response to alterations in the tissue microenvironment. The duration of SPM-dependent reprogramming is still under investigation; however, current evidence has revealed that rather than irrevocably changing macrophage state, SPM promotes a stable pro-resolving phenotype, while inflammatory challenge is contained. M2-like macrophages are effective at inducing inflammation resolution, efferocytosis, and tissue repair. However, uncontrolled or sustained M2 activation could prove detrimental: sustained activation of restorative processes may enhance generation of Transforming Growth Factor (TGF)-, extracellular matrix proteins and pro-fibrotic mediators, promoting airway remodeling and fibrosis. Ideally, SPM-based therapeutic strategies should restore macrophage plasticity, rather than facilitate unrestrained M2-biased activation [15,116].

### 5.7. Relevance to Asthma Pathophysiology

Asthma, an example of chronic airways disease, is a lung condition where there is continual inflow or stimulation from a variety of inflammatory and oxidative stressors. Asthma may affect normal synthesis of SPMs or alter their receptor activity; both of these effects can lead to decreased plasticity of macrophages, defects in efferocytosis, and prolonged airway inflammation. Restoration of SPM signaling will assist with normalizing macrophage numbers and function, reducing airway remodeling, and promoting epithelial repair. Thus, macrophage reprogramming is proposed as a key cellular mechanism through which SPMs demonstrate therapeutic effects in chronic airways disease. Table 5 below presents the multiple ways that different families of SPMs affect macrophage polarization and efferocytosis [10,69,70,110].

## 6. SPMs in Asthma Pathogenesis

SPMs are endogenous lipid mediators generated from polyunsaturated fatty acids, actively promoting inflammation resolution and tissue repair [134]. Rather than simply reducing inflammatory responses in a negative manner like conventional anti-inflammatory strategies, SPMs enhance the clearing of inflammatory cells, mediators, debris and ensure that homeostasis of tissue is re-established [69,70]. Accumulating evidence now suggests that asthma is a disease of not only overwhelming inflammation activation but also impaired inflammation resolution. Downregulation of SPM production, attenuated receptor signaling and impaired efferocytosis all contribute to chronic airway inflammation, airway hyperresponsiveness, excessive airway mucus production and structural remodeling. Hence, SPMs and their receptors have become vital regulatory components for maintaining airway immunity homeostasis and valuable therapeutic targets in treating asthma [10,70].

### 6.1. Defective Resolution in Asthma

Asthma is not a disease characterized only by inflammation activation but by an imbalance in resolution mechanisms [10,70]. Failure in the production of specialized pro-resolving mediators (SPMs) and an aberrant regulation of their respective receptor signaling is believed to be responsible for the perpetuation of airway inflammation and tissue remodeling [10,70].

In asthmatics, it has been found that lower levels of LXA, resolvins and protectins are suggestive of an impaired intrinsic ability to resolve inflammation, and there is a poor efferocytosis by macrophages that can lead to the accumulation of apoptotic cells [10,69,70]. Expression levels ofALX/FPR2 have been reported to be low in asthma [69].

The administration of SPM signaling restoring drugs in animal models has been shown to enhance efferocytosis, down-regulate inflammatory cytokine production and reduce airway hyperresponsiveness [10,70], indicating that failed resolution is not simply an outcome of airway inflammation but also a causative factor in asthma pathogenesis [10,70].

### 6.2. SPM Receptors in Airway Inflammation

SPMs mediate their biological actions through G-protein coupled receptors (GPCRs) that are expressed by airway epithelial cells, macrophages, eosinophils, neutrophils and lymphocytes. Major SPM-receptor ligands are ALX/FPR2 (LXA), ChemR23 (RvE1), GPR32 and ALX/FPR2 (RvD1), GPR18 (RvD2) and GPR37, the putative receptor for PD1, among others. SPMs activate these GPCRs, which induce inflammation resolution and immune homeostasis in the airway [10,70,75]. A decreased production of LXA and a decreased expression of ALX/FPR2 were reported in asthmatics suffering from the severe form of the disease, indicating impairment of the receptor-mediated SPM pro-resolving pathway [10,70]. These data confirm that a defective activity of SPMs receptors contributes to ongoing airway inflammation in human pathologies. In experimental models, selective activation of specific SPMs receptors has been shown to reduce inflammation. RvE1 activated the ChemR23 receptor and thus suppressed Th2 cytokines and eosinophilic inflammation, while RvD2 signaling via GPR18 decreased the airway hyperresponsiveness in asthmatic mouse models [75,112]. RvD1 acts via ALX/FPR2 and GPR32 receptors, and it polarizes the macrophages towards a pro-resolving profile, inhibiting the inflammatory cascade [112].

### 6.3. Modulation of Immune Cell Responses

SPMs act to control key innate and adaptive immune cell mechanisms that underlie the development of asthma. The eosinophil, in particular, is a main target for the inhibitory actions of SPMs in asthma, where it reduces eosinophil chemotaxis and promotes the macrophages’ clearance of dying eosinophils. This ultimately prevents further inflammation mediated by eosinophils within the airway [10,70,75]. In vitro-based studies on SPMs revealed that they suppress Th2 and Th17 cytokine production. In addition, SPMs also enhance the generation of Treg cells and suppress the dendritic cell activation/presentation of antigen. SPMS were also found to suppress neutrophil activity and ROS production [70,75]. In vivo-based animal studies revealed a reduction in eosinophils and neutrophils within the airways, decreased Th2 cytokines and attenuation of airway inflammation and lung function in animal asthma models [10,70]. It is therefore clear that SPMs coordinate numerous different cell populations to re-establish an immune balance and ensure removal of airway inflammation.

### 6.4. Airway Structural Cells

SPMs can regulate airway structural cells such as epithelial cells and airway smooth muscle cells involved in asthma pathophysiology [69]. It has been demonstrated by studies on human airway epithelial cells that SPMs diminish the burden of oxidants, inhibit the secretion of pro-inflammatory cytokines and reduce the expression of the gene mucin-associated protein MUC5AC. SPM enhances epithelial tight-junction and barrier function and thereby contributes to inhibition of allergen infiltration and chronic immune activation [135,136,137]. SPMs inhibited airway smooth muscle contraction and reduced airway hyperresponsiveness via modulation of the intracellular Ca^2+^ signal and inflammatory responses in animal models. SPMs could inhibit airway remodeling processes and reduce airway inflammation by decreasing TGF-signaling pathways, which leads to a decrease in collagen deposition. It also helps restore airway homeostasis [69,138].

### 6.5. Molecular Signaling Pathways in Asthma

Chronic inflammation in asthma is maintained by constitutive activation of signaling pathways that contribute to cytokine production, immune cell trafficking to the lungs, and excessive mucus secretion and airway remodeling. SPMs combat chronic inflammation primarily through their abilities to inhibit both NF-kB and MAPK signaling and stimulate the anti-inflammatory/pro-resolving PI3K/Akt and STAT6 pathways, respectively. Via receptor-mediated signaling, SPMs suppress the production of pro-inflammatory cytokines, increase macrophage efferocytosis and aid M2 polarization of macrophages, respectively. Many SPMs also act via the TGF/Smad signaling pathway to inhibit airway remodeling and fibrosis and facilitate tissue repair. While blocking of NF-kB is consistent among several families of SPMs, the contribution of other pathways appears receptor-dependent and context-dependent. Collectively, these pathways create a switch in the airway milieu from chronic inflammation to dynamic resolution and tissue repair [69,139,140].

### 6.6. Airway Remodeling and Fibrosis

Airway remodeling is a feature of chronic asthma and results from chronic airway inflammation and structural airway wall changes. Features of airway remodeling are goblet cell hyperplasia, basement membrane thickening, subepithelial fibrosis, smooth muscle hypertrophy and an accumulation of fibroblasts. Chronic activation of pro-fibrotic mediators, including TGF-, causes the abnormal buildup of collagen and remodeling of the extracellular matrix, leading to airflow limitation and airway hyperresponsiveness. Research using animal models indicates that specialized pro-resolving mediators (SPMs) can ameliorate airway remodeling. SPM may reduce airway remodeling by down-regulating profibrotic signaling driven by TGF-, decreasing collagen synthesis and stimulating healing mechanisms. Not only do SPMs reduce chronic inflammation, but they may also help to restore the architecture of the airways to their normal state rather than progressing towards fibrosis. Unfortunately, these anti-fibrotic mechanisms are largely derived from preclinical animal models, and more clinical work will be necessary to assess their clinical relevance in asthma [38,41,69,135]. To differentiate the translational evidence for SPM actions in asthma, the experiments are classified into the different experimental systems, from in vitro cells, to animals and humans Table 6.

## 7. Translational and Drug Development Perspectives

### 7.1. Synthetic Analogs and Structural Optimization

SPs have powerful anti-inflammatory and pro-resolving properties, and their application for therapeutic treatments has focused on developing synthetic analogs with improved pharmaceutical characteristics. Structural modification for better metabolic stability, selectivity toward the specific receptor and good drug-like properties have been performed, such as the use of fluorinated analogs, methyl ester derivatives and the introduction of cyclopropyl and benzo-analog, as well as aspirin-triggered epimers that are resistant to enzymatic activity [143]. Through these modifications, they can increase the affinity of binding to specific receptors like ALX/FPR2 and ChemR23, as well as having a better pharmacokinetic profile and tissue distribution. In this sense, the desired effects of indigenous SPs on resolution can be maintained, and at the same time enhance their translational and therapeutic potential [69,143,144]. Structural optimization is still the most practical approach to address these limitations to be translated into effective therapy for chronic inflammatory diseases like asthma.

### 7.2. Pharmacokinetics and Pharmacodynamics (PK/PD)

Understanding the pharmacokinetic and pharmacodynamic properties of SPM-based asthma therapies is fundamental for developing novel compounds. Pharmacokinetics refers to the absorption, distribution, metabolism and excretion of the drug, which dictates drug levels within target tissue. Because of the lipophilic nature of SPM, the characteristics of their distribution will have an impact on tissue penetration and access to the target receptor. Because it allows direct targeting to the airways, while simultaneously reducing exposure of other tissues, hence potential off-target effects [69,143], an inhalable-based approach is attractive. Pharmacodynamics encompasses the effects seen upon occupation of a receptor and subsequent signaling cascade. Key pharmacodynamic properties will include receptor binding affinity, potency and efficacy of an agonist, capacity for pro-resolving signaling (e.g., activation of PI3K/Akt and AMPK signaling) while inhibiting inflammatory signaling pathways. Identification of biased agonism and receptor-specific signaling profiles will assist in the development of targeted therapy with greater safety and efficacy [145,146]. A key translational aspect is also to clarify what dosage strategy would be appropriate for SPM-based therapy. Since SPM are active at such low concentrations, and have such a complex pharmacokinetic profile, the dose required for a consistent therapeutic effect, the dose, dose frequency and duration are presently unclear. These questions can only be answered with dose-ranging studies and PK/PD modeling.

### 7.3. Clinical Development Challenges

Although there is promising preclinical data supporting the use of specialized pro-resolving mediators (SPMs) in asthma, there remain several significant clinical translation hurdles. Endogenous SPMs have short plasma half-lives and are rapidly metabolized, meaning it is difficult to provide sustained therapeutic levels. Asthma is itself a heterogeneous disease (eosinophilic, neutrophilic, steroid-resistant phenotypes) that makes patient stratification and response definition difficult. There is a lack of reliable biomarkers of resolution and relatively few large, randomized controlled trials investigating SPMs, preventing adequate clinical validation. From a development perspective, there are considerable issues relating to their manufacture because of their complex stereochemistry, potential for oxidative degradation, and the need for very high purity, stability and batch lot consistency, which further complicates manufacture and therefore production costs. The regulatory pathways also remain poorly defined, SPMs being endogenous lipid mediators with no precedent for drug approval, requiring extensive data on their pharmacokinetics, safety and long-term effects, as well as efficacy based on mechanism [69,70,143,147]. The completed and in-progress clinical trials of SPMs and analogs are shown in Table 6.

### 7.4. Potential Clinical Advantages

SPM has many possible clinical uses as a treatment for asthma. Unlike traditional anti-inflammatory drugs, which suppress the immune response, SPMs promote resolution without compromising the host’s ability to fend off infection and to prevent secondary infection. SPMS may be effective as a steroid-sparing drug in severe or corticosteroid-resistant asthma. SPMs stimulate resolution of inflammation (e.g., by encouraging phagocytosis of immune cells and efferocytosis by macrophages, reducing neutrophil infiltration to the airway and the repair of the epithelial barrier). Current treatments focus on suppressing inflammation, whereas SPMs promote resolution, an area where there is currently no pharmacological intervention. The specificity of SPM and biased signaling at the receptor may permit more specific immunomodulation, which encourages repair and could potentially prevent airway remodeling. Therefore, SPMs could potentially be ideal drugs as they restore immune homeostasis rather than blocking inflammation. The main potential candidates are shown in Table 7 [69,70,139,147,148].

## 8. Current Limitations

### 8.1. Stability and Bioavailability Issues

The most significant hurdle in developing therapies based on endogenously generated SPMs is their poor biochemical stability and rapid metabolic degradation. SPMs are effective at the nanomolar range in a highly localized and temporary fashion during inflammatory resolution. These qualities are associated with an exceedingly brief half-life in circulation and poor bioavailability. Breakdown by enzymes is rapid, systemic half-life in circulation is short and highly lipophilic domains like cellular membranes and plasma proteins bind them strongly. All these features result in less effective drugs being available to reach the receptors, so it is difficult to reach the therapeutic range in vivo [69,74,77,143].

Furthermore, the oxidative and nitrosative atmosphere of asthma exacerbates this problem by increasing lipid peroxidation and reducing the production of endogenous SPMs by damaging lipoxygenases and increasing their degradation. Combined, these issues have made the systemic delivery of native SPMs very inefficient. To overcome these, stable synthetic SPMs have been produced. Many different structures were created by modifying SPMs to be metabolically stable, with examples including epimers and fluorinated SPMs, encapsulated forms or chemical protection to improve stability, bioavailability and half-life in vivo [69,74,143,155].

### 8.2. Receptor Redundancy and Cross-Reactivity

SPMs elicit their biological effects via numerous GPCRs, including ALX/FPR2, ChemR23, GPR32, GPR18, and GPR37. As a consequence of these factors, the pharmacology of these receptors can be rendered complicated: Firstly, the majority of these receptors are cognate to more than one endogenous ligand; Secondly, expression of these receptors may be species-specific; and thirdly, most of the receptors can stimulate overlapping intracellular signal transduction cascades, such as those involving the PI3K/Akt, MAPK or NF-B pathway. For example, ALX/FPR2 is a receptor for lipoxins, some resolvins, annexin A1, and SAA, and may represent a potential means by which inflammatory context-dependent or competitive signaling responses are generated. Hence, both receptor redundancy and receptor cross-reactivity may confound clinical outcomes, introduce inter-patient variability into SPM pharmacodynamics, and may also affect optimum profiles of biased agonist activity. Unfortunately, and as a result of insufficient characterization of the specificity and signaling profile of these receptors and the competition of ligands, there are still major translational limitations [74,75,156,157].

### 8.3. Limited Large-Scale Clinical Trials

Although potent pro-resolving mediator (SPM) analogs have been effective in pre-clinical asthma models and have demonstrated promising activity in early human trials, there are very few well-powered, large randomized controlled trials of the use of SPM analogs for asthma. The available data consists mainly of in vitro cell-based and in vivo animal studies, and to date, very few human trials. The human trials are few, generally small, short-term and extremely variable in design; therefore, direct comparisons between trials are unreliable and firm conclusions on long-term efficacy and safety cannot be made. It is of note that in only a few trials have objective, standardized clinical outcome measures been employed or direct comparisons made between the use of SPM analogs and conventional therapy (e.g., Steroids, biologics). The lack of Phase II and III trial data limits the feasibility of defining standard regulatory routes for the development of SPM-based therapies [70,147].

### 8.4. Lack of Standardized Biomarkers

At present, there are no validated biomarkers that will reliably and predictably predict a patient’s capability to repair tissue, that can confirm activation of SPM pathways and that are predictors of a successful treatment response in asthma. Concentrations of particular SPMs vary widely between individual patients due to levels of intake of omega-3 fatty acids in the diet, a patient’s metabolic state, the pre-existing inflammation burden, as well as genetic polymorphisms of receptors and enzymes in the biosynthetic pathways [69,77]. This variability between patients means that stratifying patients and monitoring changes to treatment can be difficult Lipidomic analysis by mass spectrometry is capable of measuring SPM profiles accurately, but is a very costly and technically demanding procedure and is not yet a standard clinical tool, and hence there is a clear need to develop and validate clinically relevant, robust and reproducible biomarkers for patient stratification, monitoring and evaluation of the pharmacodynamic response in further clinical trials. The absence of such tools hinders attempts to establish the basis of personalized approaches for the treatment of asthma based on SPM therapy [69,70,77].

### 8.5. Disease Heterogeneity in Asthma

There is not one single disease of asthma; rather, the diseases are composed of varying endotypes of asthma, which are then defined by multiple different immune signatures, cytokine and airway injury patterns. The endotypes recognized to date are: Type 2-high or eosinophilic, neutrophilic, steroid-resistant and mixed inflammatory phenotypes. Each group of asthma endotypes will be distinct in terms of its production of specialized pro-resolving mediators (SPM) and their receptor expression and signaling patterns. Thus, it can be suggested that the deficiencies in pathways of resolution in eosinophilic asthma will not necessarily be the same as those deficiencies in neutrophilic or steroid-resistant asthma, and it seems unlikely that there can be one SPM-based therapeutic platform that will be effective in all individuals who have asthma [69,135].

### 8.6. Manufacturing and Formulation Challenges

Asthma’s therapeutic target is the airway; therefore, inhalation is the ideal target for delivery of the specialized pro-resolving mediators (SPMs). However, the lipid-based nature of SPMs renders inhalation an extremely challenging area of formulation science. SPMs are poorly water-soluble, making their delivery via conventional inhalation vehicle formulations impossible. Polyunsaturated structures in SPMs will likely have low stability during storage due to oxidative damage, and the dose of the medicine will change. Delivering effective aerosol formulations is technically challenging, as uniformity and physical stability of particle size are required for correct deposition in the lung, and nebulization can induce chemical degradation by light, air and mechanical stress. Stable and reproducible inhalation formulation is difficult to manufacture [69,143].

### 8.7. Dosing and PK Uncertainty

This pharmacokinetic inter-patient variability of novel specialized pro-resolving mediators poses a significant challenge when attempting to establish dosage regimens of SPMs. Differences in lipid metabolism, enzyme expression and activity and inflammatory state among individuals can result in dramatically different SPM absorption, distribution, metabolism and clearance, thus resulting in a significantly varied system exposure and therapeutic response among patients. Due to the naturally short half-life of most SPMs, sustained systemic levels may be best achieved by frequent doses or specialized long-release delivery mechanisms, thus adding another layer of complication to dose strategy and patient compliance; these factors clearly indicate a need for well-designed dose–response studies in the development of an effective and reliable dosing strategy for SPM-based treatment regimens [69,77].

## 9. Future Directions

### 9.1. Precision Medicine and Biomarker-Guided Therapy

It is anticipated that the application of SPM-based therapies in clinical practice will be more endotype-specific rather than generalizable to all asthmatics. Future precision medicine applications might include the stratification of patients based on inflammatory phenotypes (eosinophilic, neutrophilic, mixed or steroid-refractory asthma), receptor expression signature and basal endogenous SPM levels. Biomarker-guided approach, including lipidomic signature or evaluation of SPM receptor responsiveness, may aid in the identification of those patients who will best respond to therapy and in the evaluation of response to therapy. A combined ‘resolution index’ incorporating numerous biochemical and molecular markers would be most indicative of a patient’s pro-resolving capacity. Validation of the use of these biomarkers is critical to better select patients, optimize treatment response, and facilitate the transition of SPM-based therapies into clinical practice [69,70,147,158].

### 9.2. Strategies to Enhance Therapeutic Effectiveness

In the future, the further development of SPM-based therapy specifically for use in combination with established asthma treatments will be examined. Combination treatments using SPM analogs with corticosteroids, biologics or leukotriene modifiers could offer a dual approach by not only encouraging active resolution of inflammation but also maintaining inflammatory pathway inhibition, consequently leading to potentially reduced steroid dependence. It is possible that SPM-based therapy could also offer a route for treating treatment resistance seen in severe and steroid-resistant asthma, as these treatments target native pathways that current anti-inflammatory approaches do not affect directly. Further development may also see receptor-specific SPM agonists that possess greater pro-resolving and tissue-repairing actions than endogenous SPMs. Combined, these could offer an advanced and enhanced approach in a treatment regimen [69,139,155].

### 9.3. Delivery and Formulation Improvements

Emerging delivery technologies may serve to mitigate many of the limitations posed by specialized pro-resolving mediator (SPM) substances, such as instability, accelerated degradation, and low bioavailability. Techniques using liposomal formulations, nanoparticle delivery, and inhalable systems could protect SPMs from degradation through oxidative and metabolic routes and enhance drug retention and efficacy. In addition, inhalation-based delivery strategies used for asthma provide for targeted pulmonary deposition and increased localized concentrations with lower systemic exposure, further highlighting the future promise of SPMs as therapeutic agents for asthma through improved delivery systems [108,143,159].

## 10. Conclusions

It is increasingly recognized that asthma is more than an increase in inflamed tissue. Asthma is viewed as a failure of resolution of inflammation rather than simply the persistence of ongoing immune stimulation. This conceptual transition from viewing asthma as a consequence of an accumulation of excessive immune activity to the simultaneous failure of an endogenous mechanism of immune resolution has been associated with a shift in airway pathophysiology. By integrating knowledge from structural biology, enzymatic generation of specialized pro-resolving lipid mediators (SPMs), receptor pharmacology and macrophage phenotype reversal, we will illustrate that SPMs act as an endogenous modulator of airway immunity. The failure of SPM synthesis, receptor activation and convergent signaling via SPMs are associated with prolonged expression of NF-B and MAPK, a deficiency in efferocytosis, persistent activation of either Th2 or non-Th2 inflammation and progressive airway remodeling. Rather than being an immunosuppressant in the traditional sense, SPMs modulate separate aspects of an overarching resolution program that are capable of reprogramming the macrophage phenotype (to an M2 and non-inflammatory state), repairing damaged epithelial structures, mitigating unwanted fibrocyte activation pathways and re-establishing an immune milieu at the local level. New discoveries in the area of receptor modeling and biased agonism reveal that SPM receptor signaling plasticity (ALX/FPR2, ChemR23, GPR18, GPR37) is context-dependent and will be vital in facilitating rational drug design to selectively enhance pro-resolution transcriptional networks while limiting unwanted pro-inflammatory activation of these same networks. SPM-based drugs offer promising future avenues for therapy for those with steroid-resistant asthma and other disorders associated with chronic oxidant stress, PI3K/Akt pathway activation and glucocorticoid resistance. In contrast to attempts to suppress the symptoms of inflammation, these drugs target an immune defect responsible for perpetuating disease, and in so doing, they offer new strategies for management. Although challenges remain regarding pharmacokinetics and the longevity of an introduced drug, recent successes in synthetic analog development, targeted nanotechnology delivery systems, and the molecular engineering of the receptors themselves offer realistic translational potential for the therapy of chronic airways diseases. The combination of structural pharmacology, systems-based immunology and targeted biomarker identification/characterization will eventually answer the question of whether or not SPM-based therapies will become new therapeutic targets.

## Figures and Tables

**Table 1:** Macrophage Phenotypes in Asthma.

Phenotype	Key Markers	Functions in Asthma	Pathological Role	References
M1 (Classically Activated)	iNOS, TNF-α, IL-1β, IL-6, CXCL9, CXCL10	Produce pro-inflammatory cytokines; antimicrobial activity; drive Th1/Th17 responses	Exacerbate airway inflammation, epithelial damage and oxidative stress; contribute to steroid resistance	[15,51,52,53]
M2a (IL-4/IL-13–induced)	Arginase-1, YM1, Fizz1, CD206	Promote tissue repair, fibrosis and eosinophil recruitment	Airway remodeling, fibrosis, goblet cell hyperplasia	[43,54,55,56]
M2b (Immune complex/TLR–induced)	IL-10, TNF-α, IL-6, CCL1	Regulatory macrophages balancing inflammation and repair	Can sustain chronic inflammation if uncontrolled	[54,57]
M2c (IL-10, TGF-β–induced)	MerTK, CD163, IL-10, TGF-β	Anti-inflammatory, immunoregulatory, tissue remodeling	May promote airway fibrosis and persistent remodeling	[43,55,57]
M2d (Tumor-associated/angiogenic)	VEGF, IL-10, CCL18	Promote angiogenesis, tissue remodeling	Contribute to airway vascular remodeling and chronicity	[43,54,58]

**Table 2:** Summary of Preclinical and Clinical Evidence Supporting Specialized Pro-Resolving Mediators (SPMs) in Asthma.

SPM Subclass	Preclinical Studies	Major Preclinical Findings	Clinical Studies	Major Clinical Findings	Contradictions/Limitations	References
Lipoxins (LXA_4_, LXB_4_)	BALB/c mice; OVA-induced allergic asthma; BALF and lung tissue; human bronchial epithelial cells	Reduced eosinophilia, airway hyperresponsiveness (AHR), mucus production; enhanced macrophage efferocytosis	BALF, sputum, plasma from severe eosinophilic asthma patients	Reduced LXA_4_ levels associated with severe disease and impaired resolution	Variability among BALF, sputum, and serum measurements; influence of corticosteroid use	[10,15]
Resolvins (RvD1, RvE1)	BALB/c mice; OVA-induced asthma; BALF, lung tissue; murine alveolar macrophages; human epithelial cells	Reduced Th2 cytokines, eosinophilic inflammation, AHR; enhanced efferocytosis and M2 polarization	BALF, sputum, plasma from mild-to-severe asthma patients	Lower resolvin levels correlate with disease severity and reduced lung function	Differences among asthma phenotypes, obesity status, and sample sources	[10,49,92,93]
Protectins (PD1)	Murine LPS-induced lung injury and viral airway inflammation models; BALF and lung tissue; epithelial cell cultures	Reduced leukocyte infiltration, improved epithelial repair, increased IL-10 production	BALF, sputum, serum from severe asthma cohorts	Reduced PD1 levels linked with impaired epithelial repair and exacerbation risk	Limited clinical studies; inconsistent associations across phenotypes	[10]
Maresins (MaR1, MaR2)	BALB/c mice; OVA-induced asthma; BALF, lung tissue; murine macrophages	Enhanced efferocytosis, reduced collagen deposition and airway remodeling, restored epithelial integrity	BALF, sputum, plasma from severe asthma patients	Lower MaR1 levels associated with persistent inflammation and airway remodeling	Emerging clinical evidence; limited patient numbers	[10,49,94]
Aspirin-Triggered SPMs (AT-LXA_4_, AT-RvD1)	OVA-induced asthma and fibrosis models; BALF, lung tissue; macrophage cultures	Reduced eosinophilia, fibrosis, inflammatory cytokines; improved resolution signaling	Limited human studies; inflammatory tissue and blood samples	Evidence of enhanced resolution signaling following aspirin exposure	Limited asthma-specific clinical data; variability among aspirin-responsive phenotypes	[10,49,95]

**Table 3:** Comparative Signaling and Pharmacological Features of SPM Receptors.

SPM	Receptor	Primary G-Protein Coupling	Key Downstream Pathways	Biased Signaling Features	Functional Outcome	References
LXA_4_	ALX/FPR2	Gαi/o	PI3K/Akt, IκB stabilization	Preferential NF-κB inhibition over ERK activation	Reduced neutrophil infiltration	[101]
RvE1	ChemR23	Gαi	Akt, ERK modulation	Partial agonism; anti-inflammatory bias	Enhanced efferocytosis	[102]
RvD1	GPR32	Gαi	STAT6, NF-κB suppression	Selective activation of pro-resolving transcription	M2 polarization	[103]
RvD2	GPR18	Gαi	Rac1 activation, PI3K/Akt	Promotes phagocytosis without MAPK overactivation	Bacterial clearance + resolution	[104]
PD1	GPR37	Likely Gαi	SMAD2/3, ERK modulation	Cytoprotective signaling bias	Epithelial protection	[105]
MaR1	Putative GPCR	Gαi/AMPK-linked	AMPK, PPARγ	Metabolic reprogramming bias	Tissue repair	[106]

**Table 5:** Effects of Different SPM Families on Macrophages.

SPM Family	Receptor(s)	Effect on Macrophage Polarization	Effect on Efferocytosis	Key Preclinical/Clinical Evidence	References
Lipoxins (LXA_4_, LXB_4_)	ALX/FPR2 (primarily for LXA_4_)	Promote M2 phenotype, inhibit pro-inflammatory cytokine production; protect macrophage viability by activating PI3K/Akt and ERK/Nrf2	Stimulate non-phlogistic phagocytosis of apoptotic cells by macrophages; improve clearance in acute inflammation models	LXA_4_ reduces inflammation and fibrosis, preserves tissue function in models of lung injury and renal disease	[127,128,129,130]
Resolvins (E-series, D-series)	ChemR23 (RvE1), GPR32, GPR18	Shift macrophages from M1 to M2 phenotype; reduce TNF-α, IL-6; increase IL-10 production	Enhance macrophage-mediated clearance of apoptotic cells; restore defective efferocytosis in pathologies	Reduce airway inflammation in asthma models; promote microbial clearance in infections	[75,131,132]
Protectins (PD1, PDX)	GPR37 (putative)	Shift macrophages from M1 to M2 phenotype; reduce TNF-α, IL-6; increase IL-10 production	Accelerate clearance of apoptotic neutrophils; enhance epithelial repair via TGF-β/SMAD signaling	Attenuate inflammation and tissue damage in viral airway inflammation models.	[75,132,133]
Maresins (MaR1, MaR2)	Unknown GPCR(s)	Promote mitochondrial oxidative phosphorylation; induce M2 markers Arg1 and IL-10; inhibit NF-κB signaling	Enhance efferocytosis via MerTK upregulation; accelerate clearance of apoptotic debris	Significantly reduce fibrosis and improve lung function in asthma models	[85,112]

**Table 6:** Evidence Supporting Specialized Pro-Resolving Mediator (SPM) Actions in Asthma Across Experimental Systems.

Evidence Level	SPM	Model/System	Key Findings	References
Cell culture	RvD1	Human macrophages	Enhanced efferocytosis, increased IL-10 production, promotion of pro-resolving phenotype	[48]
Cell culture	LXA_4_	Human airway epithelial cells	Reduced IL-8 release and NF-κB activation	[10]
Animal study	RvD1	Ovalbumin-induced murine asthma	Reduced eosinophilic inflammation and airway hyperresponsiveness	[141]
Animal study	MaR1	Murine asthma model	Reduced collagen deposition, enhanced efferocytosis, improved lung function	[10]
Human study	LXA_4_	Severe asthma patients	Lower endogenous LXA_4_ levels associated with impaired resolution	[15]
Human study	RvD1/SPM pathway	BALF and plasma samples from asthma patients	Evidence of defective pro-resolving mediator pathways in severe disease	[142]

**Table 7:** Therapeutic Modalities of Specialized Pro-Resolving Lipid Mediators (SPMs) and Analogy in Asthma.

SPM or SPM-Analog	Targeted Mechanism	Preclinical/Clinical Evidence	Delivery Method	References
Lipoxin A4 (LXA4) and Analogy	ALX/FPR2 receptor activation; inhibition of eosinophil chemotaxis and activation; macrophage M2 polarization; anti-fibrotic effects	Reduced airway inflammation, bronchial hyperresponsiveness, and tissue remodeling in murine asthma models; Limited stability in humans, ongoing analytical development	Nasal spray or inhalation formulations; lipid-stable analog designed for enhanced bioavailability	[148,149,150]
Resolvin E1 (RvE1) and D-Series Resolvins	Bind ChemR23, GPR32, and GPR18; suppression of pro-inflammatory cytokines; promotion of M2 polarization and efferocytosis	Demonstrated reduced eosinophilic inflammation, airway remodeling, and improved lung function in preclinical asthma models	Inhaled aerosol delivery and systemic administration in experimental models	[151,152]
Protectin D1 (PD1) and Derivatives	Modulate immune cell recruitment; promote epithelial integrity; activate TGF-β/SMAD pathways for repair	Suppresses airway eosinophilia, reduces cytokine storm in viral asthma exacerbations; protective effects in murine allergen challenge	Intranasal or intravenous routes; potential for a synthetic stable analog	[152]
Maresin 1 (MaR1) and Maresin Conjugates (MCTR)	Enhance macrophage efferocytosis via MerTK; mitochondrial metabolic reprogramming; inhibition of fibroblast activation	Improved airway hyperresponsiveness, fibrosis reduction, and inflammation resolution in preclinical models	Liposomal formulations, inhalation, and synthetic analogy development	[82,153,154]

## Data Availability

No new data were created or analyzed in this study.
